# Supplementary figures and images for: Immunity onset alters plant chromatin and utilizes EDA16 to regulate oxidative homeostasis
Source: PLoS Pathog. 2021 May 20;17(5):e1009572. doi: 10.1371/journal.ppat.1009572 (PMC8171942; doi:10.1371/journal.ppat.1009572)

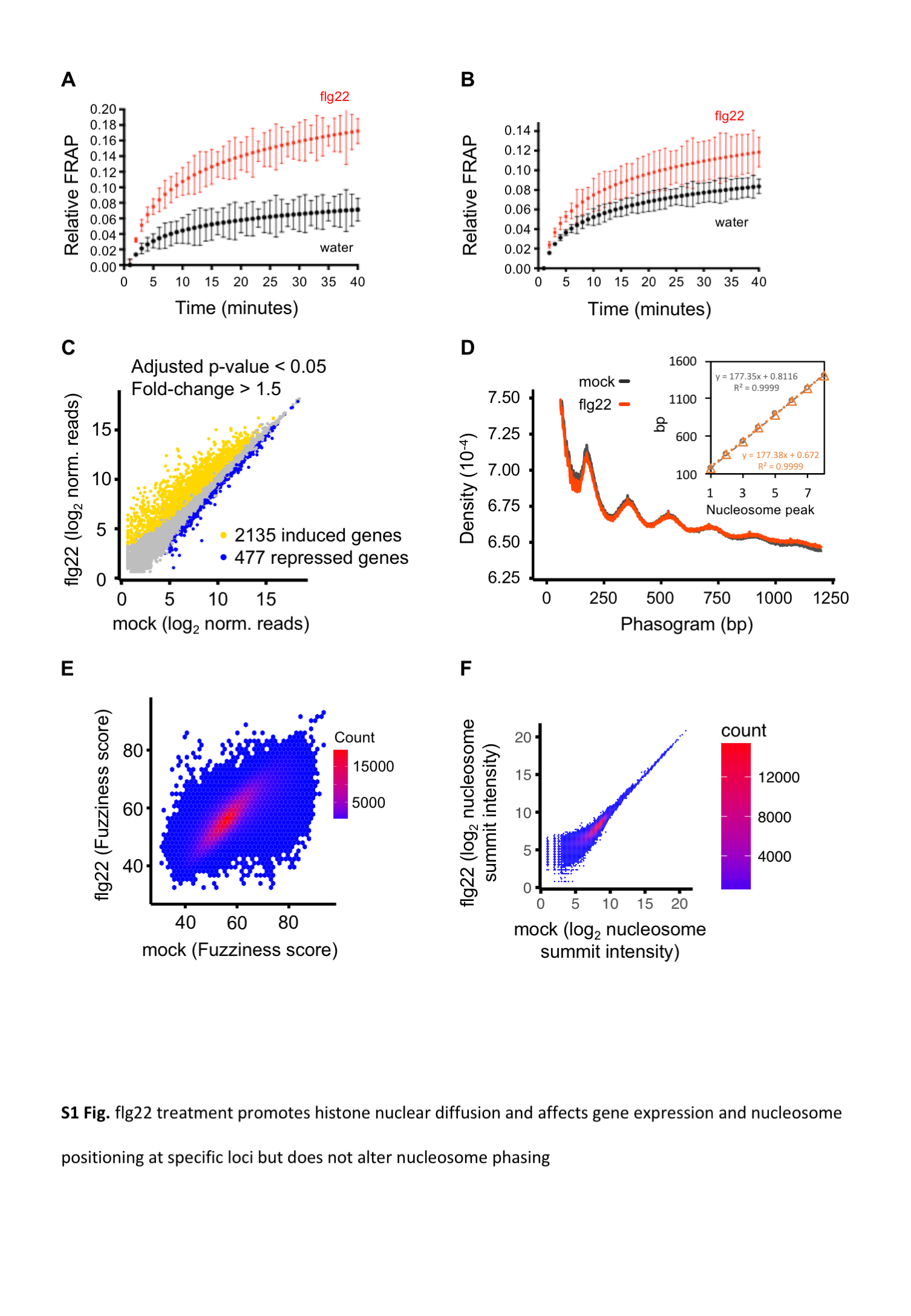

Supplement: S1 Fig — (A) FRAP data collected from seedling leaf tissue H2B-GFP in Col-0 or (B) transient expression in Nicotiana benthamiana adult leaves. The tissue was exposed to water or 100 nM flg22 for 1 hour before imaging. Data points are averages of at least 8 nuclei for each condition. Error bars represent standard error of the mean. (C) flg22-regulated genes. RNA-seq gene expression scatter plot showing Differentially Expressed Genes (DEGs, adjusted p-value < 0.05, fold-change > 1.5) on 2-week-old Arabidopsis seedlings (Col-0) following elicitation with 100 nM flg22 compared with mock; induced (yellow), unaltered (grey) and repressed genes (blue). (D) flg22 elicitation does not change the average genomic nucleosome phasing. Nucleosome phasogram of Col-0 plants following 100 nM flg22 treatment (red) and control (black). On top right corner linear correlation fit between nucleosome peak and base pairs (bp). Red, treatment (slope = 177.37 bp/nucleosome) and black control (slope = 177.37 bp/nucleosome). (E) Nucleosome fuzziness. Analysis of nucleosome fuzziness at mock state (x-axis) compared with 100 nM flg22 treatment (y-axis) using Dynamic Analysis of Nucleosome Position and Occupancy by Sequencing (DANPOS, FDR < 0.01). (F) Nucleosome summit intensity. Analysis of nucleosome peak at mock state (x-axis) compared with 100 nM flg22 treatment (y-axis) using DANPOS (FDR < 0.01). (TIF) [file ppat.1009572.s001.tif]

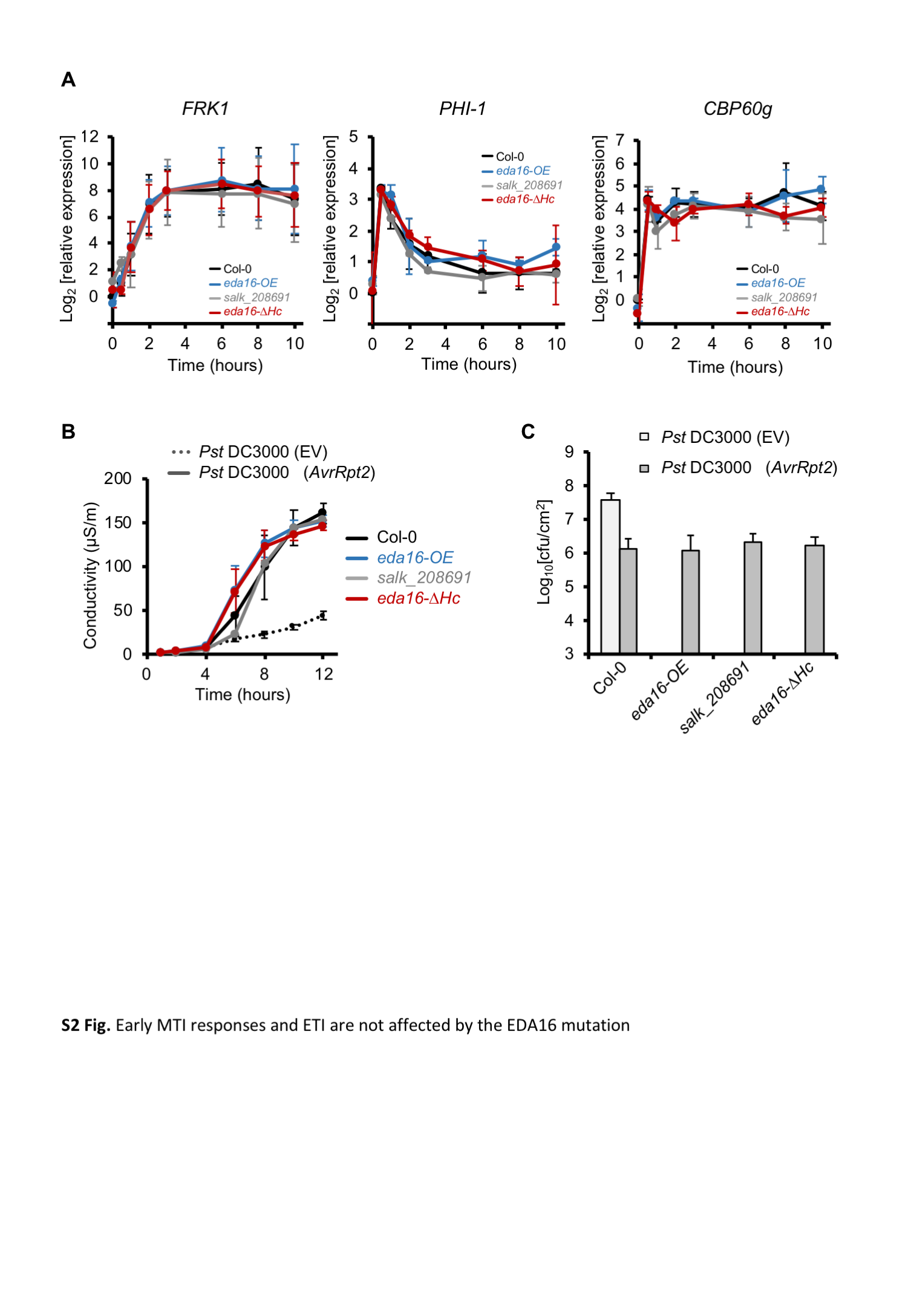

Supplement: S2 Fig — (A) Accumulation of FRK1 (left), PHI-1 (middle) and CBP60g (right) transcripts was assessed by qPCR in 2-week-old Col-0 (black) eda16-OE (blue), eda16 line SALK_208691 (grey) and eda16-ΔHc (red) seedlings elicited with 100 nM flg22. Values are average of three biological repeats ± SE presented as fold induction compared with Col-0 mock-treated sample at time 0. (B) and (C) ETI responses in eda16 mutants. 5-week-old Col-0, eda16-OE, salk_208691, and eda16-ΔHc plants were syringe-infiltrated with Pst DC3000 EV or Pst DC3000 avrRpt2. For Ion leakage leaf disks were collected and kept in sterile water. Conductivity measurements (microsiemens per meter) were taken from the solution at different times as indicated (B). Bacterial colony forming units were determined 3 days post-infection (C). Error bars represent standard deviation (n = 6) and the experiment has been repeated 3 times with identical results. Differences were not statistically significant (two-sided T-test) between Col-0 and the eda16 mutants. (TIF) [file ppat.1009572.s002.tif]

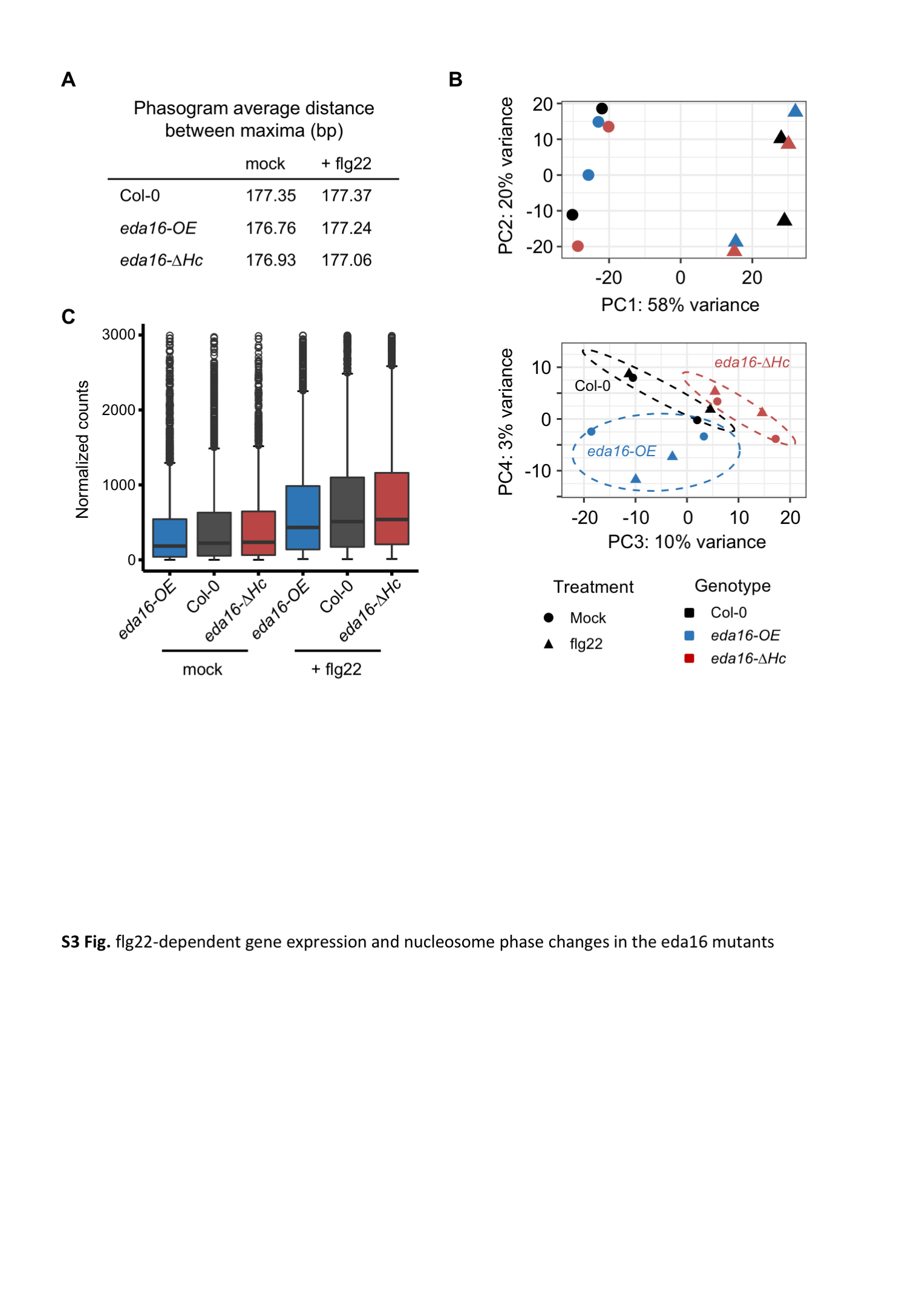

Supplement: S3 Fig — flg22-dependent gene expression and nucleosome phase changes in the eda16 mutants. (A) flg22 elicitation does not change the average genomic nucleosome distribution in the eda16 mutants. Nucleosome phasogram of Col-0, eda16-OE and eda16-ΔHc plants before (mock) and after elicitation with flg22 (100 nM). (B) EDA16 affects flg22-regulated genes. Principal component analysis (PCA) of RNA-seq normalized read count data reveals a greater difference in gene expression between flg22- and mock-treated plants (principal components 1, PC1 and PC2, accounting between the two for near ~80% of the variance) than between different genotypes (clustered by PC3 and PC4, accounting between the two for ~13% of the variance). (C) Gene count distributions in Col-0 and eda16 mutants following elicitation with flg22. Normalized count distributions are displayed as boxplots for Col-0, eda16-OE and eda16-ΔHc for mock-treated or elicited with flg22 (100 nM) plants. (TIF) [file ppat.1009572.s003.tif]

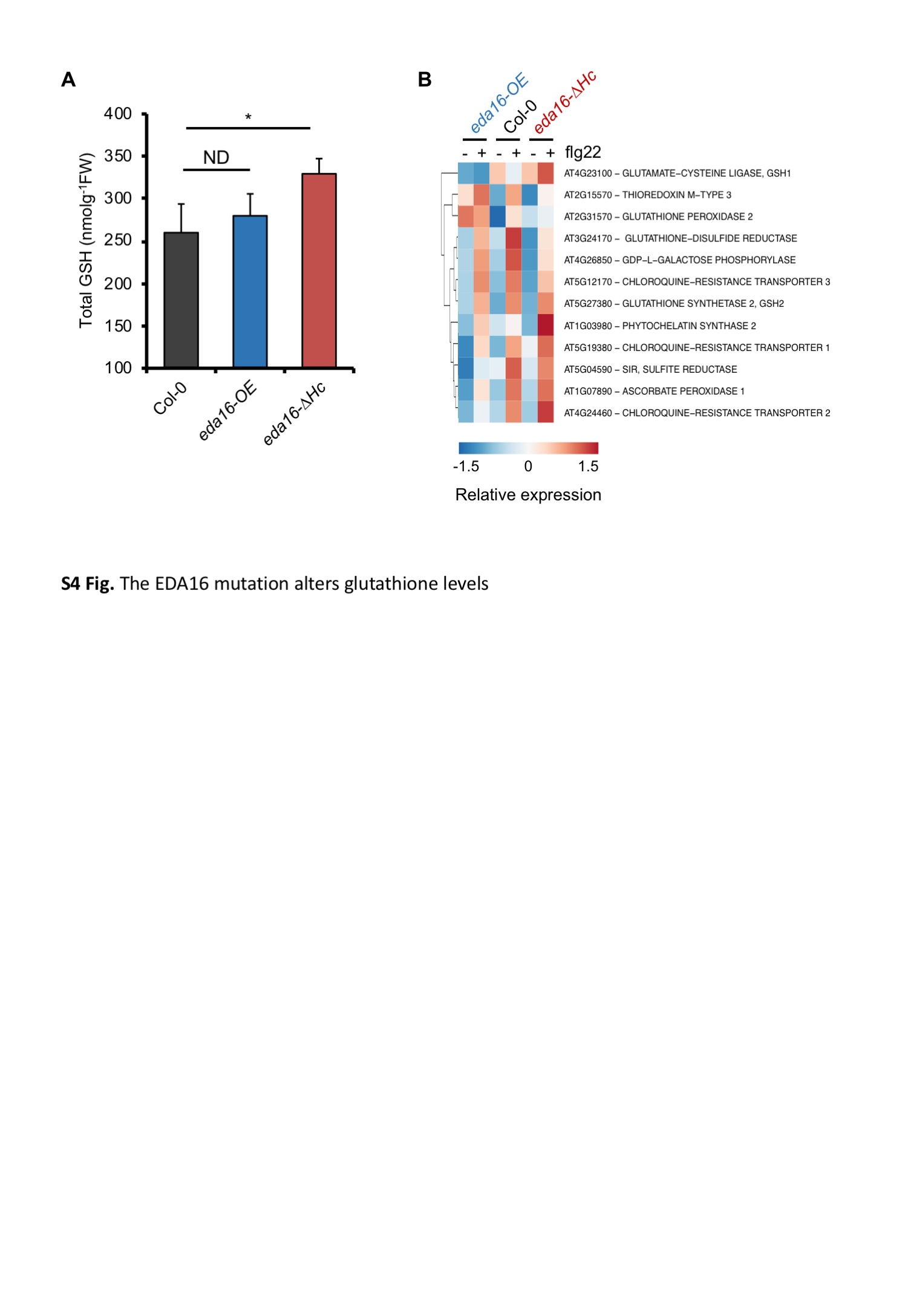

Supplement: S4 Fig — (A) The eda16-ΔHc mutant has elevated glutathione (GSH). Basal total glutathione (GSH) levels were determined in 3-week-old Col-0, eda16-OE and eda16-ΔHc plants. Error bars represent standard deviation, n = 3. Statistical differences are indicated (two-sided T-test p-values: * < 0.05). (B) EDA16 regulates the expression genes involved in glutathione production. Gene expression heatmap for genes involved in glutathione production between Col-0, eda16-OE and eda16-ΔHc plants 2h after elicitation with 100 nM flg22. (TIF) [file ppat.1009572.s004.tif]

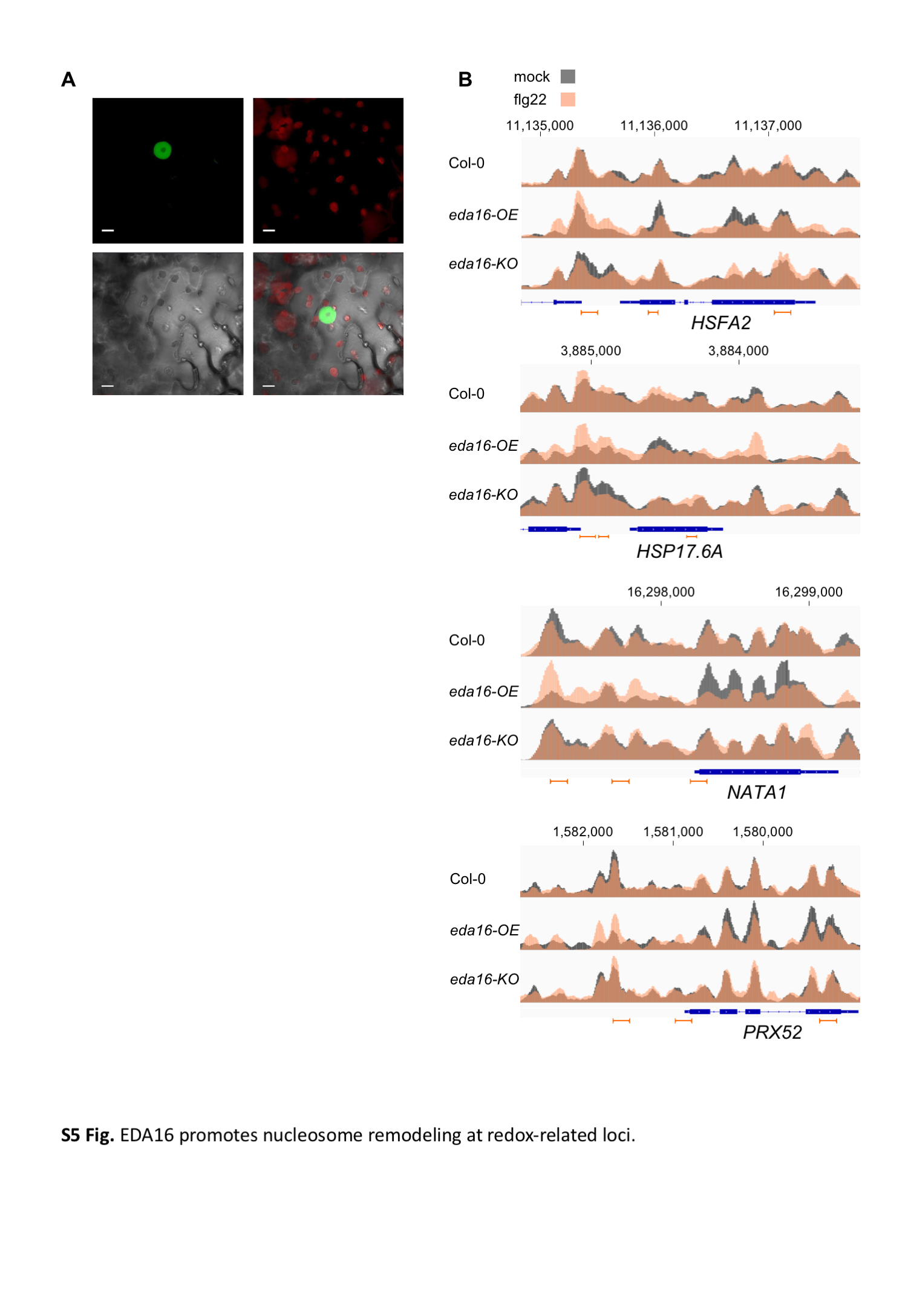

Supplement: S5 Fig — (A)EDA16 is localized in the nucleus. Confocal localization of 35S::EDA16-YFP construct. (B) EDA16-mediated nucleosome repositioning. IGV image of MNase-seq reads over HSFA, HSP17.6A, NATA1, and PRX52 loci for Col-0 (top), eda16-OE (middle), and eda16-ΔHc (bottom) as indicated. Tracks for mock (grey) and flg22 (pink) conditions are overlaid. Primers used in Fig 5E indicated below their respective IGV gene track in orange. (TIF) [file ppat.1009572.s005.tif]
