# Supplementary material for: Immunity onset alters plant chromatin and utilizes EDA16 to regulate oxidative homeostasis
Source: PLoS Pathog. 2021 May 20;17(5):e1009572. doi: 10.1371/journal.ppat.1009572 (PMC8171942; doi:10.1371/journal.ppat.1009572)
Supplement: S1 Table — (DOCX) [file ppat.1009572.s006.docx]

# S1 Table. Sequencing mapping statistics for MNnase-seq (Supports Figs 1-5)

| **Sample** | **Total Reads** | **Mapped %** | **Chromatin coverage*** |
| --- | --- | --- | --- |
| Col-0 mock BR1 | 55,567,851 | 98.95 | 28.2x |
| Col-0 mock BR2 | 59,195,856 | 99.25 | 31.2x |
| Col-0 flg22 BR1 | 48,149,590 | 99.09 | 21.6x |
| Col-0 flg22 BR2 | 64,897,733 | 99.27 | 35.6x |
| eda16-OE mock BR1 | 52,961,022 | 98.57 | 26.5x |
| eda16-OE mock BR2 | 65,622,002 | 99.25 | 28.4x |
| eda16-OE flg22 BR1 | 38,682,055 | 98.99 | 18.4x |
| eda16-OE flg22 BR2 | 59,800,378 | 99.23 | 29.9x |
| eda16-∆Hc mock BR1 | 51,628,939 | 98.99 | 21.7x |
| eda16-∆Hc mock BR2 | 57,838,632 | 99.13 | 31.1x |
| eda16-∆Hc flg22 BR1 | 52,727,373 | 98.81 | 28.7x |
| eda16-∆Hc flg22 BR2 | 69,623,372 | 99.15 | 35.2x |

* Chromatin coverage excludes reads mapped to chloroplast and mitochondrial DNA
