# Supplementary material for: Immunity onset alters plant chromatin and utilizes EDA16 to regulate oxidative homeostasis
Source: PLoS Pathog. 2021 May 20;17(5):e1009572. doi: 10.1371/journal.ppat.1009572 (PMC8171942; doi:10.1371/journal.ppat.1009572)
Supplement: S2 Table — (DOCX) [file ppat.1009572.s007.docx]

# S2 Table. Differentially positioned nucleosomes (DPNs) between flg22- and mock treated samples (Col-0, *eda16-OE* and *eda16-∆Hc*) detected with any of DANPOS parameters, summit, point or nucleosome fuzziness.

| **DANPOS parameter** | **Col-0** | ***eda16-OE*** | ***eda16-∆Hc*** |
| --- | --- | --- | --- |
| Summit | 7015 | 10709 | 5001 |
| Point | 6844 | 10591 | 4854 |
| Fuzziness | 21920 | 19380 | 17819 |
| Any | 27102 | 28796 | 21386 |
