# Supplementary material for: Immunity onset alters plant chromatin and utilizes EDA16 to regulate oxidative homeostasis
Source: PLoS Pathog. 2021 May 20;17(5):e1009572. doi: 10.1371/journal.ppat.1009572 (PMC8171942; doi:10.1371/journal.ppat.1009572)
Supplement: S3 Table — (DOCX) [file ppat.1009572.s008.docx]

# S3 Table. Differentially positioned nucleosomes (DPNs) between flg22- and mock treated samples mapped to protein-coding gene regions (promoters; including -1000 bp from TSS, TSS; including 180 bp, +/- 90 bp from TSS, gene bodies; from TSS to TES and promoters + gene bodies).

| **DANPOS parameter** | **Gene regions** | **Col-0** | ***eda16-OE*** | ***eda16-∆Hc*** |
| --- | --- | --- | --- | --- |
| Summit | Promoters | 2553 | 3018 | 1874 |
|  | TSS | 323 | 462 | 342 |
|  | gene bodies | 1287 | 3300 | 814 |
|  | genes+promoters | 3643 | 5878 | 2563 |
| point | Promoters | 2532 | 2994 | 1807 |
|  | TSS | 327 | 449 | 332 |
|  | gene bodies | 1237 | 3262 | 787 |
|  | genes+promoters | 3580 | 5837 | 2469 |
| fuzziness | Promoters | 5598 | 4109 | 5190 |
|  | TSS | 1174 | 804 | 1230 |
|  | gene bodies | 8791 | 7950 | 6782 |
|  | genes+promoters | 12555 | 10837 | 10595 |
| any | Promoters | 7097 | 6315 | 6230 |
|  | TSS | 1390 | 1167 | 1462 |
|  | gene bodies | 9283 | 9791 | 7143 |
|  | genes+promoters | 13938 | 13802 | 11596 |
