# Supplementary material for: Immunity onset alters plant chromatin and utilizes EDA16 to regulate oxidative homeostasis
Source: PLoS Pathog. 2021 May 20;17(5):e1009572. doi: 10.1371/journal.ppat.1009572 (PMC8171942; doi:10.1371/journal.ppat.1009572)
Supplement: S4 Table — (DOCX) [file ppat.1009572.s009.docx]

# S4 Table. Chromatin remodelling ATPase T-DNA insertion mutants and primers for genotyping and cloning *EDA16* cDNA.

| **AGI** | **Gene Name** | **T-DNA insertion line** | **Sequence forward (LP)** | **Sequence reverse (RP)** |
| --- | --- | --- | --- | --- |
| At1g03750 | SWI2, CHR9 | SAIL_1265_E08 (*chr9-1*) | ATCTTTCCGGAGCAGTAGCTC | GAAGCCCTGTGGTAGTTTTCC |
|  |  | SALK_064383C | CAGAACGGAAAAACTTGTTTTTG | TCGGAATCTCTCCAATTGATG |
| At1g05120 |  | SAIL_757_C04 | AACAACCAAATCTCGTCATGG | ACAATGGGACGATTAGGGATC |
|  |  | SAIL_451_E01 | TTGCAGCAAGTCATTTTACCC | TTGGCTTGGCCACATACTAAC |
| At1g05490 | CHR31 | SAIL_739_F04 | TTGAGCTTTGCAAATCCTCAC | AGAGAAGGGCATGAAGAAAGC |
|  |  | SALK_204501C | CAGGTCTTGATGGCTCTTCAG | GGAGAAGAAATCAGGTCCCAG |
| At1g08060 | MOM1 | SAIL_610_G01 | ACAATGCAGGAGCAAACACTC | GGAAAGGAGATACTTCACCGG |
|  |  | SALK_141293 (*mom-2*) | ACGAGAATTCATGAATCACGC | CTGCTCACCAAGAATCTGGAG |
| At1g08600 | CHR20, ATRX | SALK_025687C | GGAGGTAATGGAGGTGAAAGC | GTCAAGCTCAGATGTTCCAGC |
|  |  | SALK_024609C | TGTAGGATGGCGTGATCTAGG | TGTTGCTGCTGAAATGATGAC |
| At1g11100 |  | WiscDsLoxHs079_12B | ACACTTCAATTCCAGGCAGAG | TCTAATGTTTTTGGTCAAACCTG |
|  |  | SALK_113298C | ACGCTCACAAGGGAATATGTG | ATTTTCATGCAGACTTGCAGG |
| At1g48310 | CHR18 | WiscDsLox435D8 | TTCCCCACAAAGACTTGTGAC | GATTGCAAGAAAAATGGCAAG |
|  |  | SAIL_154_H02 | TTCCCCACAAAGACTTGTGAC | CTCTCACATTACCTTCGTCGC |
| At1g50410 | CHR28, FRG2 | SALK_057016 (*frg2-1*) | CATAAGGGGAACCGAAAGAAG | GCTGAAGGTTGCATTTCTGAC |
|  |  | WiscDsLox324G06 (*frg2-2*) | AAGCCATTTGACCATTCTGTG | TCTTCGGTTTGTGCATTTTTC |
| At1g61140 | EDA16 | SAIL_40_F09 (*eda16-OE*) | CTTTGCACATGGTTGTTTGTG | CGAAATTCAGAAGTAGACGCG |
|  |  | SALK_107256.28.05.x | TCGATCCACATGTCTAATTCC | GTGAATCCTTGAGCTCGTCAG |
|  |  | SALK_208691 | CGAGAAGAGGGTGATCACAAG | AACGCTTGTTGTATGTCCCAC |
|  |  | SAIL_1156_D12C1 | CGAAGCTCGTTCTCATGAATC | AACAGTGCATTTATGAACGCC |
|  |  | SAIL_735_G06 (*eda16-∆Hc*) (*frg4-1*) | AAAGTGTCCAAGCGAGAGACTC | ATGAGGGTATGAGCGTTGATG |
| At2g02090 | CHR19, ETL1 | SALK_054130C | ACCATCCAACGAAACACACTC | AAAGCTCTTTTCGAAGATCCG |
|  |  | SALK_069014C | CAAGTTGCTGCATGACATCAG | TTGAATCTGTGATGATCGCAG |
| At2g18760 | CHR8 | SALK_000799C | TACCGTTTCAACAAAACCAGC | TCTTTGACGAAACCAGTTTCG |
|  |  | SAIL_381_A07 | TGGGTACCATTTCTGTGCTTC | CTGATTCCTGTGGGATTATGC |
| At2g21450 | CHR34 | SALK_014697 | TTTGCCAAAACAATTTCAAAAC | AAATCATGCAAAACCCATTTG |
|  |  | SALK_059100C | TTATGGGAAACAAAAAGACGC | CTGGACAGAGAAGCAGACACC |
| At2g44980 | CHR10, ASG3 | SALK_084703C | TGGAGTTGGCAGTTATGAACC | GAAGCTCAATAAACGACGTCG |
|  |  | SALK_095969C | TTCATACAGCCAGGACACGTAC | CTCGAAACGGTAGCGTATGAC |
| At3g16600 |  | SALK_067458C | CAGGAGGTAGATCCTTTTCGG | CATGACTGATCTTCTAGCCCG |
|  |  | SAIL_1254_D05 | ATTTCTCTGTCCAAGCCAACC | CGTCGAGATCGTTTTACCAAG |
| At3g19210 | CHR25, RAD54 | SALK_205160C | AAACAATGGAATACGCGTTTG | AGTCATAGGAAGAGGAAGCGC |
|  |  | SAIL_667_A08 | CTTTTAAGCTGCATGCAGAGG | GGAGTTTAGGTGATGCTGCAC |
| At3g20010 | CHR27, FRG1 | SAIL_1271_F06 | AGATCGATTCAAACGGTGTTG | TGAAACCCACTACAAAGCCAC |
|  |  | SALK_063135 (*frg1-2*) | ATTTTTCTGAGGTGCATGGTG | TCATCGTTCTCATCCTCATCC |
| At3g54460 |  | SALK_008164 | ACGGTTGATCACAATTTCTGG | TGTTCCGGAATTCTCAACATC |
|  |  | SALK_065434C | CATCATGATTTCCTGGACTGG | TCGGACTCGAAACAGAAGAAG |
| At4g31900 | CHR7, PKR2 | SALK_118921C | CAAACTGTTTGAATTCCTCTCG | CATCTTGTACGCTCCTCCTTG |
|  |  | SALK_115303C | TTCTGATTTTTCAACCGATGG | GGGGAGGAGTATCTGGTGAAG |
| At5g07810 |  | SALK_133371.43.40.x | AGGTGTTGGGAAGATTAACGG | TTTCTCAATTGACGAAGGCAC |
|  |  | SALK_113907.31.15.x | ATGCTATGGCAAGTCATCTGC | TGGCAAAAATATAATGCCTCG |
| At5g18620 | CHR17 | SALK_139387C | TGCCATTAACTTACCGCAGAG | TATAACGGAACGGCCTTATCC |
|  |  | SALK_080144C | ACCTGCGTTTGTTACAAGGTG | TTACCTGCATGAGTACAGGGG |
|  |  |  |  |  |
| Genotyping SALK lines | | LBb1.3 | ATTTTGCCGATTTCGGAAC |  |
| Genotyping SAIL lines | | LB3 | TAGCATCTGAATTTCATAACCAATCTCGATACAC | |
| Genotyping SALK lines | | RB1.2 | CTCCTTCAACGTTGCGGTTCTG | |
| Genotyping Wisconsin lines | | p745_WiscDsLox | AACGTCCGCAATGTGTTATTAAGTTGTC | |
| Cloning EDA16 cDNA | | EDA16_cDNA_F1 | AAAAAGCAGGCTCCACCGGTGAGGAAGGTTCAAT | |
|  |  | EDA16_cDNA_R1 | AGAAAGCTGGGTCTCATGAATCAGCCATAAAC | |
|  |  |  |  | |
