# Supplementary material for: Immunity onset alters plant chromatin and utilizes EDA16 to regulate oxidative homeostasis
Source: PLoS Pathog. 2021 May 20;17(5):e1009572. doi: 10.1371/journal.ppat.1009572 (PMC8171942; doi:10.1371/journal.ppat.1009572)
Supplement: S5 Table — (DOCX) [file ppat.1009572.s010.docx]

# S5 Table. Primers for qPCR and ChIP-qPCR

| **AGI** | **Name** | **Sequence (5' to 3')** |
| --- | --- | --- |
| AT1G04820 | αTUB F | TACACCAACCTCAACCGCCT |
|  | αTUB R | TGGGGCATAGGAGGAAAGCA |
| AT1G49240 | ACT8-F | CCAGTGGTCGTACAACCGGTA |
|  | ACT8-R | TAGTTCTTTTCGATGGAGGAGCTG |
| AT5G26920 | CBP60g-F | AAGAAGAATTGTCCGAGAGGAG |
|  | CBP60g-R | GGCGAGTTTATGAAGCACAG |
| AT1G61140 | EDA16-5 F | GGTGAGCAGCCTGTGGAAAC |
|  | EDA16-5 R | CCCAACCCCACTCCGTATCC |
|  | EDA16-mid F | CGCATTGTCATGGATGGCCC |
|  | EDA16-mid R | AGGCGCACATTCTCCAGTCT |
|  | EDA16-3 F | TCTCGGACTGAACATGGTGGC |
|  | EDA16-3 R | GTGAGACTGTCGGCTTCCCTT |
| AT2G19190 | FRK1 F | ATCTTCGCTTGGAGCTTCTC |
|  | FRK1 R | TGCAGCGCAAGGACTAGAG |
| AT1G35140 | PHI-1 F | TTGGTTTAGACGGGATGGTG |
|  | PHI-1 R | ACTCCAGTACAAGCCGATCC |
| AT4G34270 | TIP41 F | GTTGGTGCCTCATCTTCGCC |
|  | TIP41_R | GAACTGGCTGACAATGGAGTGT |
| AT5G05340 (*PRX52*) | qPRX52_F | CCACGGCAGCTTCTTTCGAC |
|  | qPRX52_R | GAGTCAGTAGAGCCGCCGTT |
|  | chPRX52_F1 | attaatctgtgtaggggtggcg |
|  | chPRX52_R1 | ccaaatggtccacacacatcaatg |
|  | chPRX52_F2 | cactttagaacatcccttcacgtct |
|  | chPRX52_R2 | GAGAGGAGGTTGGGGCAAGAG |
|  | chPRX52_F3 | CCACGGCAGCTTCTTTCGAC |
|  | chPRX52_R3 | GAGTCAGTAGAGCCGCCGTT |
| AT2G39030 (*NATA1*) | qNATA1_F | GCAGTGAAGTTGGGTGTCGG |
|  | qNATA1_R | GAAGTGCATCGCCAGTGAGC |
|  | chNATA1_F1 | tcgacaaatttggactaagagacgt |
|  | chNATA1_R1 | tcgttagcacaaaatagttagccga |
|  | chNATA1_F2 | tggtatggtcgtgcgttgtct |
|  | chNATA1_R2 | ggtcaccgatcatctctccca |
|  | chNATA1_F3 | agcagccttagaagcaagtaaca |
|  | chNATA1_R3 | GGTCGGTGAAGTCTCTGGCAC |
| AT2G26150 (*HSFA2*) | qHSFA2_F | AGCTTGGGGACTATGGAGGAGA |
|  | qHSFA2_R | GTCCCAATCCAAAGGCGAACC |
|  | chHSFA2_F1 | aattgtgcagcaggtggagac |
|  | chHSFA2_R1 | ttaatgtgggccgccagaaaa |
|  | chHSFA2_F2 | GCTTAACGAAACAGGGCCACC |
|  | chHSFA2_R2 | CAAGAAACCACCGTGTCCGTC |
|  | chHSFA2_F3 | AGCTTGGGGACTATGGAGGAGA |
|  | chHSFA2_R3 | GTCCCAATCCAAAGGCGAACC |
| AT5G12030 (*HSP17.6A*) | qHSP17.6A_F | TGGAGAGGAGGATGGGGAAGT |
|  | qHSP17.6A_R | ACACACCGTCATTACAAGCCG |
|  | chHSP17.6A_F1 | tgactcatgaatcctctgcgtttg |
|  | chHSP17.6A_R1 | tcaggcccaagaccaattcgt |
|  | chHSP17.6A_F2 | acgaattggtcttgggcctga |
|  | chHSP17.6A_R2 | cgtcttgatcgttggagaagtgt |
|  | chHSP17.6A_F3 | TGGAGAGGAGGATGGGGAAGT |
|  | chHSP17.6A_R3 | ACACACCGTCATTACAAGCCG |
|  |  |  |
|  |  |  |
