# Supplementary material for: Immunity onset alters plant chromatin and utilizes EDA16 to regulate oxidative homeostasis
Source: PLoS Pathog. 2021 May 20;17(5):e1009572. doi: 10.1371/journal.ppat.1009572 (PMC8171942; doi:10.1371/journal.ppat.1009572)
Supplement: Attachment — Submitted filename: 2021.docx [file ppat.1009572.s024.docx]

We thank the Editor and the Reviewers for their comments which helped us to improve the manuscript. In the revised manuscript we have edited the text and figures according to the reviewer’s recommendations, we have also added new data in figure 3,5 and S1, and we have included additional data in figure S5 and in datasets. We hope these revisions will sufficiently address the concerns of the reviewers.

Specific responses to reviewer comments in red:

Reviewer #1- major issues

*The title states that “EDA16 regulates oxidative homeostasis”. The results* p*resented are, however, entirely consistent with an indirect role of EDA16 on oxidative homeostasis, which may or may not be mediated by the group of genes affected in eda16 mutants. Demonstrating direct interaction of EDA16 with the suspected target genes by chromatin immunoprecipitation using an antibody against EDA16 is be an essential experiment to justify such a conclusion*.

We thank reviewer 1 for this important suggestion. We believe that our data supports a model for a direct interaction which we have now tested by chromatin immunoprecipitation assays (ChIPs). We have now included these experiments in the revised manuscript (lines 344 - 351, Fig 5E, Fig S5, lines 422 – 423 and corresponding material and methods). In these experiments, we were able to demonstrate binding of EDA16 to 3 of the 4 potential target genes selected for the assay.

We believe that these experiments have strengthen the conclusions drawn increasing the impact of the paper. We therefore thank the reviewer for it.

*The abstract claims that a novel feedback mechanism has been revealed (l. 30). Again, in my opinion this is not supported by the data presented. I fail to see the “feedback” element (the moderate transcriptional upregulation of EDA16?). This statement needs to be clarified (and possibly backed by additional experimentation) or toned down.*

We appreciate the comment of the reviewer 1. We have now performed a time course of induction after flg22 treatment for several genes (*PRX52*, *HSFA2*, *HSP17.6A* and *NATA1*) associated with redox homeostasis detected by our combined RNA-seq/MNase-seq analysis (Fig 5A). The gene expression supports our previous results, with opposite trends for the two mutants with Col-0 in between. Importantly, the results (except *PRX52* for which the gene expression decrease was not observed within the time points chosen in Col-0 wild type) would lend support to the idea of EDA16 role in gene repression after 2-4 hrs, favouring the notion of a negative feedback mechanism. These new results have been added to Fig 5D (see below) lines 335 - 344 and lines 419 - 422 in the revised manuscript.

In addition, following the reviewer’s recommendation, the statement in the abstract (lines 30 - 32) has been rephrased.

*Similarly, the last sentence of the abstract “framework to prevent protracted activation of immunity” is an overstatement. While this is an interesting idea, I wonder whether the effects of eda16 on a group of 10 genes that, as far as I can see, have not previously been recognized to be central to the MTI response, can constitute a general mechanism that would warrant to call it a “framework”. Moreover, while the prevention of protracted gene activation by chromatin remodelling is an interesting hypothesis, the manuscript does not show any timecourse of gene expression that would indicate that genes are active for longer in eds16 mutants and that this causes the enhanced resistance*.

Again, we appreciate the comment of the reviewer 1, and would like to clarify that with this statement we would like to refer to all chromatin remodeling ATPases identified in this study. In order to add the necessary clarity, we have modified the text accordingly (Lines 35 - 37).

In addition, as explained above, we have now performed a time-course experiment (see new figure 5D, presented above). This new experiment demonstrates the gene induction for *HSFA*, *NATA1* and *HSP17.6A* at 2 hours after flg22 treatment, followed by a down-regulation at later timepoints, which is particularly delayed for the compromised mutant *eda16-∆Hc*, whereas exacerbated for the *eda16-OE* mutant. These results, together with the gene expression pattern for *EDA16* (Fig 3A, B and F), would support the notion of a negative feedback loop mechanism.

We hope that now the text is clear in our meaning.

Reviewer #2- major issues

*In the present manuscript, authors have shown that EDA16 (member of Ris1 subfamily) is involved in nucleosome repositioning in plant defense. MNase-seq and RNA-seq were performed in Col-0 and 2hrs flg22 treated Col-0. The similar experiments were also performed in eda16-OE and eda16-∆Hc to identify DPNs (which could either be enriched nucleosomes or depleted showing either increase in occupancy or decrease) over promoter or genic region (Line 254-268, 139-144), the contrasting DPNs were also observed in eda16-OE and eda16-∆Hc. Thus, authors concluded the involvement of EDA16 in chromatin remodeling during plant defense. Considering EDA16 is ATPase chromatin remodeler, it would be nice if they clearly classify DPNs into enriched and depleted and try to correlate their RNAseq data with those enriched/depleted nucleosomes. It will help in clearly depicting the role EDA16 in chromatin remodeling*.

We thank the reviewer for this interesting comment. We understand the appeal of this suggestion since such a clear-cut classification has the potential to reveal in detail EDA16 mode of action. However, high resolution results have mostly been reported for *in vitro* reconstitution assays ( [Krietenstein et al., 2016](https://www.ncbi.nlm.nih.gov/pmc/articles/PMC5240917/) ) or in the case of the highly-ordered yeast genome ( [Udugama et al., 2011](https://www.ncbi.nlm.nih.gov/pmc/articles/PMC3028646/) ). For *in vivo* data, it is often necessary to compound many observations together in order to detect a phenomenon such as nucleosome phasing ( [Li et al., 2014](https://onlinelibrary.wiley.com/doi/full/10.1111/tpj.12499) ). For this reason, we have presented the average nucleosome plots and differential occupancy plots of flg22-induced genes with DPNs (1,142), showing that *eda16-OE* presents an extreme trend to deplete nucleosomes on the gene body of induced genes while increasing nucleosome density towards the promoter (Fig 4B).

DANPOS recognises nucleosome perturbations in three categories, peak summit intensity (relating to a well-positioned nucleosome that changes peak intensity across treatment/mutants; relates to a significant proportion of the nuclei sequenced losing that particular nucleosome); fuzziness (nucleosomes may not have a well-defined position across cells, leading to broader peaks) and finally, point distance (nucleosomes may have experienced an observable shift from their reference position when re-mapped to a treatment). These three parameters are not necessarily independent and they are not mutually exclusive, which yields complex data. We have tried to address the exact comment by compiling a list of DPNs (according to the only DANPOS parameter directly referred to occupancy, i.e. peak height, to define enriched nucleosome when peak height is statistically different according to DANPOS, then we annotated whether it is enriched or depleted). With this list, which is much shorter than if all three DANPOS parameters are considered (since each parameter present different variation see Fig S1 E-F), we made a classification, as requested. Unfortunately, the loss of quality, perhaps due to analysis restrictions, makes it difficult to draw meaningful conclusions. In general, these results are similar to what has been already presented in Fig 4, Fig S3 and Tables S2 and S3 as well as Dataset 7, mainly that *eda16-OE* has a notoriously different chromatin remodelling landscape from Col-0 (and *eda16-∆Hc*). Therefore, in order avoid complicating the message unnecessarily, we felt compelled to leave out this analysis.

Originally, we had endeavoured to classify the changes experienced in flg22-regulated genes by using a k-means clustering algorithm (addressed by Fig 2 in contrast to Fig 1B), where we find that flg22-induced genes, display a complex behaviour, showing different nucleosome patterns for different groups of genes. It has been proposed that actively expressed genes tend to present a reduced nucleosome density across the gene body ( [Liu et al., 2015](https://www.ncbi.nlm.nih.gov/pmc/articles/PMC4510002/) ). However, not much is known about how this estate may be achieved when transitioning from lower expression towards higher expression levels. Our data could be interpreted in a model where EDA16 is involved in this nucleosome remodelling transaction, pushing or shifting nucleosomes away from the gene body towards intergenic regions. Our new ChIP data suggest, that this may be the case for the genes studied (Fig 5E and S5B). Nevertheless, there is no clear pattern arising from EDA16 activity on specific nucleosomes.

*To ascertain the role of EDA16 in emodelling of DPNs, they should also perform ChIP to confirm physical association of EDA16 around DPNs.*

We thank reviewer 2 for this important suggestion. We have now performed the experiment suggested. Please see the answer to Reviewer 1, comment 1.

Reviewer #3 – major comments

*Reviewer 3: The authors infer a model in which EDA16 dampens the expression of flg22 induce genes at the later timepoints. This is based on the observation that EDA16 is expressed relatively late after elicitation. If this is the case, it would make sense for the authors to test a number of flg22 and EDA16 responsive genes in a time course, assessing whether their expression drops in an EDA16-dependent manner. Testing a representative set of genes would be enough for that.*

We thank reviewer 3 for the constructive comment. We have now performed this experiment. Please see answer to Reviewer 1 comment 2 and Fig5D.

Reviewer #1: Minor Comments:

*- In the Col wild type analysis (Fig. 1) I wonder whether DEGs are enriched in DPNs and if so, whether this is statistically significant, i. e. whether proportionately more genes that are differentially expressed show changes in nucleosome structure than would be expected by chance. It looks a bit like 50% of all genes have DPNs after treatment and 50% of DEGs have DPNs (as rough estimate). This is exactly what you would expect is the two were unrelated effects.*

We appreciate the importance of this observation and we have carried out the probability calculations advised by the reviewer by computing the hypergeometric probability of two sets of known sizes (2,135 and 13,938 for induced genes and 477 and 13,938 for repressed genes) to have an overlap such as found or greater in a population of 27,418 protein coding genes. The results suggest (see figure below) that the overlap between flg22-induced genes and genes with DPNs is unlikely (p-value < 0.01), but the overlap with flg22-repressed genes is within expected values for such numbers.

(B) Part of figure 1: Venn diagram illustrating the overlap between genes with at least one DPN (grey), flg22-induced genes (yellow), and flg22-respressed genes (blue). Most significant GO terms found for the intersection groups with the TopGO package using as a control set all Arabidopsis protein coding genes (Fisher Exact Test, p-value < 0.01). Below, we show the results of the hypergeometric test as well as simulations: Induced DEGs with DPNs 1,142 (red dashed line) are over-enriched (hypergeometric p-value < 0.01) compared to theoretical (black line) and simulated (histogram bars) overlap between induced DEGs (2,135) and genes with DPNs (13,938) (left). However, repressed DEGs with DNPs (242, red dashed line) are as expected from random sampling or theoretical distribution (right).

These results have been included in the main text lines 142 – 144.

We believe there is an important role for chromatin remodelling in the regulation of the onset of immunity (and specifically MTI). Towards further illustrating this point and increasing the evidence presented in the manuscript, we have added experimental fluorescence recovery after photobleaching (FRAP) data showing at the single cell level an alteration in the diffusivity of histone H2B when the tissue has been exposed to flg22. In these experiments, we explored the effect of flagellin (flg22) on nucleosome remodelling using histone H2B-GFP FRAP as a proxy for nucleosome dynamics. These new results included in lines 108 – 113 and supplementary figure S1 and associated material and methods.

- The more relevant control group for this test in enrichment would be a pool of all the clusters except cluster 3. Naively, I would expect a lot of defense-related genes (such as the DEGs) to have WRKY binding sites in their promoters.

We thank the reviewer for the suggestion. We have now performed the enrichment analysis and found that in these conditions, when comparing cluster 3 promoters relative to the remaining flg22-responsive genes, we find no significant TF binding site enrichment. The lack of enrichment could indicate that there is no relationship between the nucleosome depletion and transcription factors. However, it is also possible that certain genes require both events (nucleosome remodeling followed by TF binding) for efficient gene induction, as some transcription factors are precluded from binding nucleosomal DNA ( [Zhu et al., 2018](https://www.ncbi.nlm.nih.gov/pmc/articles/PMC6173309/) ). Since without actual binding data it is hard to speculate which binding sites are *bona fide* or relevant to the transcriptional event and which could be permanently occluded, in consequence, we have preferred to remove these results from the manuscript (lines 169 – 172).

Additional comments:

-Several authors are not listed in the Authors Contributions list.

We thank the reviewer for spotting this error. We have now corrected the list.

-EDA16 should be spelled out at first mentioning (l. 28 or title)

We thank the reviewer for flagging this issue. We have now corrected the abstract (addressed in lines 29 – 30).

-l. 54: “relies”, -l. 222: “EXPRESSOR”

We thank the reviewer for spotting these typos, that we have now corrected on respective lines (now lines 54 and 233).

Reviewer #2: Minor Comments:

b. In line 114, author should also provide the pvalue (statistical significance) for the length (177 bp) between the nucleosome peaks.

We thank the reviewer for this constructive comment. The nucleosome dyad-to-dyad distances were estimated from a linear regression based on the first 8 peaks inferred from the phasogram analysis (Fig S1D, insert; see below) according to [Valouev et al. (2011](https://www.ncbi.nlm.nih.gov/pmc/articles/PMC3212987/)). We have now performed a paired t-test for the first 8 peaks comparing thus the differences of values for mock and treated samples (p-value inserted in lines 121 – 126 of the main text).

In addition, we now provide the *r*^2^ value for the linear regression in supplementary Fig S1D insert.

2. Author has performed MNase-seq in order to identify the nucleosomes positioning. The authors should provide the table for MNase-seq data regarding number of million paired-end read generated and coverage on the Arabidopsis genome.

We apologize for having missed this information. We have now added it to lines 121 – 126 and Supplementary Table S1. We also provide the NCBI link to access the raw data (GEO accession number GSE149654).

3. In line 124, the three parameters compared by DANPOS: nucleosomes fuzziness, summit intensity or point location. What they are representing should be provide either in introduction or in results.

We appreciate the comment. We have now added the required information to lines 132-135.

4. In Fig2A, authors have performed k-mean clustering to classify the 1142 flg22-induced genes and then identify motifs enriched in cluster3. The similar type of analysis should also be done on the 242 flg22-repressed gene.

Originally, we focused on the induced genes due to their considerably higher number that could render data analysis such as k-means classification sufficiently accurate. Furthermore, as explained above, the overlap between repressed genes and genes with DPNs is not enriched. In other words, the number of repressed genes with DPNs were found to be very close to values expected from overlaps of random sets of the same sizes (Fisher Exact Test, p-value > 0.01). Therefore, this suggests that targeted chromatin remodelling may not be a leading feature of gene repression, but perhaps a consequence.

To better clarify this point along the manuscript we introduced changes in lines 160 – 164.

5. The authors have chosen 20 chromatin emodelling ATPase mutants and then spray inoculated them with a Pseudomonas syringae pathovar tomato DC3000 (Pst DC3000). Further, they have chosen EDA16 on the basis of FC since in EDA16, the FC was highest and showed resistance phenotype. Since, with the advancement in the plant age there is tremendous change in the transcriptome profile of the plants. The author should provide the logical reason for the comparison since, the FC identified from RNA-seq data at 2-week-old plants while the experiment was performed at 5- week old plants.

We were interested in dissecting the effect due to MTI triggering (avoiding the additional effects observed when using a bacterial infection, such as those of effectors, ETS, and so on), for which we followed the standard procedure of studying the effect of flg22 on seedlings grown in sterile conditions ( [Navarro et al., 2004](https://www.ncbi.nlm.nih.gov/pmc/articles/PMC514144/) ). On the other hand, most studies with the pathogenic bacteria *Pseudomonas* *syringae* DC3000 use adult plants following the Staskawicz’s lab reliable method ( [Whalem et al., 1991](https://www.ncbi.nlm.nih.gov/pmc/articles/PMC159978/) ). In fact, landmark papers have previously combined these two techniques successfully ( Zipfel et al., 2004 ). Based on the homogeneous expression of *EDA16* across developmental stages (see below), and based on the similar induction observed both with flg22 at the seedling stage and at the adult stage with DC3000 (Fig 3A-B) and because we do not see any developmental differences in the mutants (Fig 3G), we believe our data still represent an important contribution. Finally, the redox phenotype described in adult plants recapitulates the effect captured by the transcriptomic and MNase-seq combined analyses, validating our approach.

Furthermore, the developmental map for *EDA16* expression suggests that (other than seeds and senescent leaves) the gene expression is sustained across developmental stages. From BAR ( <http://bar.utoronto.ca/efp/cgi-bin/efpWeb.cgi?dataSource=Developmental_Map&modeInput=Absolute&primaryGene=AT1G61140&secondaryGene=At3g27340&override=&threshold=1345.96&modeMask_low=None&modeMask_stddev=None&gene_alias1=EDA16&gene_alias2=> )

6. In fig 3B, authors have shown that expression of EDA16 is 3 hours following infection with Pst DC3000. It will be appropriate, if authors also provide the qPCR data of the intermediate time points also.

We have now performed a longer time course analysis during DC3000 infection and added stats as requested. Early time points can be followed by flg22 induction and later points by the DC3000 infections. This is in accordance with previous studies where gene expression after bacterial infection showed no difference between DC3000 and mutants of DC300 lacking a type III secretion system (*hrpA*) at early times points ([Lewis et al., 2015](https://www.ncbi.nlm.nih.gov/pmc/articles/PMC4682296/) ).

7. In line 290-300, authors have identified few genes (21genes) having change in the expression levels between the two mutants as well as with the Col-0. With that, these genes also have distinct nucleosome dynamics. All the performed analysis is the result obtained from the data generated from the sequencing platform. The authors should also provide experimental validation from the same.

We appreciate the reviewer’s comment and we agree on the necessity of this validation. We have now performed a time course analysis with qPCR for *PRX52*, *HSFA2*, *HSP17.6A* and *NATA1*, as described above. Please see response to Reviewer 1 comment 2 in the Part II – Major Issues section.

8. In fig 3H, authors have shown the log10(cfu/cm2 ). The result obtained showed the comparison when inoculated with DC. The authors should cross-check once their data as their error bars are almost overlapping while the pvalue obtained is “ ** < 0.01 “.

We thank the reviewer for noticing. We have checked the result and found it to be correct according to our calculations**:**

|  | LOG10 | LOG10 | LOG10 | LOG10 | LOG10 | LOG10 | MEAN | SD | T-test |
| --- | --- | --- | --- | --- | --- | --- | --- | --- | --- |
| Col-0 | 7.7118 | 7.5315 | 7.2788 | 7.4624 | 7.6752 | 7.8388 | 7.583 | 0.200 |  |
| *eda16-OE* | 7.7782 | 8.1761 | 7.9542 | 7.7970 | 8.0212 | 7.8084 | 7.923 | 0.158 | **0.00855** |

9. Author should perform following small modification:

a. Use “*” where necessary in the figure to show the statistical significance of the value like in fig 3A, 3E.

We thank the reviewer for this observation. It has been now addressed in Fig 3.

b. Similarly, in entire text, please use the similar format for a particular word. Like in some places pval is written while at other place pvalue is written.
c. Line 127, capitalize the legend number of h,i,j.

We thank the reviewer for this observation. It has been now addressed along the text.

Reviewer #3: Minor Comments:

To improve the MS, I would like for the authors to consider the following points:

- It would be useful for the authors to state how similar their flg22 transcriptome datasets agrees with the literature. Is there significant overlap between studies or differences as well?

We acknowledge that it is an important question to validate genomic data. Over 80 % of our DEGs are described as such by a recent study using the same timepoint of 2 hours and same sequencing platform: RNA-seq by Illumina ( [Birkenbihl et al., 2017](http://www.plantcell.org/content/29/1/20#sec-17) ). Even with older analyses, (though these datasets tend to contain less DEGs microarray analysis, and they focus on 1 hour-treated samples) we find around 50 % of the genes described as flg22-regulated ( [Navarro et al., 2004](https://www.ncbi.nlm.nih.gov/pmc/articles/PMC514144/) , Zipfel et al., 2004 ). Importantly, all datasets (except the smallest one containing only 354 genes) recognised *EDA16* as a DEG in the conditions studied. We have now incorporated these findings to the main text (lines 120 – 121).

- The authors look at nucleosome occupancy, responsible for DEG and directed by EDA16. It would be of interest to see to what extent, other remodelling factors are under the control or are affected by MTI and EDA16. Is there cross-talk between regulators? Are there distinct ‘immune sectors’ that are controlled by EDA16?

According to our RNA-seq data (Table 1), in Col-0 background (wild type), only *EDA16* and AT1G11100 (*FRG5*) are affected by flg22 after 2 hours. This result was echoed when searching for genes differentially regulated in *eda16* mutants due to flg22 (Fig. 5). Our data suggests that a sector of genes related to redox homeostasis and oxidative stress are particularly affected in *eda16* mutants (Fig 5 and Fig S5).

- Now that the role of EDA16 has been established and it has emerged as an important regulator, one critical question is: how is EDA16 expression regulated? It is clear that WRKY TFs play important roles in MTI, so the obvious question is as to whether cognate responsive elements can be found in the Eda16 promoter.

We thank the reviewer for this insightful comment. Indeed, as speculated by the reviewer, through transcription factor binding motif analysis (TRANSFACT public dataset) we found putative sites for MIB, ABI and WRKY transcription factors including WRKY18 in the *EDA16* promoter. This became even more relevant when we could confirm in publicly available data, that WRKY18 presents a ChIP peak within the *EDA16* promoter (peak at coordinates 22,541,567 – 22,541,909; chromosome 1, within 1000 bp upstream of *EDA16* TSS; [Birkenbihl et al., 2017](http://www.plantcell.org/content/29/1/20#sec-17) ). Furthermore, *wrky18* mutation showed *EDA16* was no longer found as a differentially expressed gene in response to flg22 treatment ( [Birkenbihl et al., 2017](http://www.plantcell.org/content/29/1/20#sec-17) ), suggesting a role of WRKY18 in upregulating *EDA16* expression during flg22 perception, and further stressing the role of *EDA16* in plant immunity. These findings have now been added to the main text in lines 241 – 249.

Similarly, and to identify possible feedback loops, it would be interesting to know whether EDA16 itself could be a target and experiences DNP. Analyses of the promoter regions in the OE and mutant lines (for which data is available) may offer yet more interesting clues.

We appreciate the reviewer’s comment. However, the only clear differences in nucleosome occupancy during flg22 induction of *EDA16* can be observed for the *eda16-OE* mutant and they correspond to the general pattern observed in other induced genes (nucleosome increase over promoter peaks with some depletion towards the 3’ end). We could not detect a distinctive pattern on the wild type or defective mutant *eda16-∆Hc* (see figure below). To avoid diluting the message, we have chosen not to include this in the main text.

(A) IGV image of MNase-seq reads over *EDA16* locus for Col-0 (top), *eda16-OE* (middle), and *eda16-∆Hc* (bottom) overlapping mock (grey) and flg22 (pink) conditions. (B) Nucleosome occupancy difference between flg22-treated and mock-treated Col-0 (black), *eda16-OE* (blue), and *eda16-∆Hc* (red) for *EDA16* locus.

- In the MS, a number of datapoints, that could prove to be revealing, are not described. For example, in Figure 4, a set of genes appear to be positively regulated by EDA16 as these genes are highly induced during OE (in both mock and flg22 treated plants) and repressed in the mutant. What kind of proteins do they encode?

We thank the reviewer for this constructive comment. We apologize for not being sufficiently clear. We provide all sequencing raw data (GEO accession number GSE149654) and analysed RNA-seq data as part of supplementary tables (Datasets S1, S8 and S10). We have now combined this information for all DEGs in at least one of the genotypes to include it in the supplementary material data (Dataset S14). On this occasion, we preferred to remain focused on those genes that we could confidently relate to a flg22 response (Fig 5), over genes that may be differentially expressed due to other effects of the mutations or related to potential roles of *EDA16* outside MTI.

- A weaker aspect of this MS relates to the changes in redox state, attributed to EDA16 action. While the data, demonstrating changes, is convincing, the induction of GST and changes in redox status could be due to downstream effects (induction/repression of genes controlled by EDA16). While I appreciate the difficultly untangling cause and effect relationships, the authors should be careful to not over-interpret their results in that regard.

We appreciate the comment and we have sought to soften the claims along the manuscript (changes were made in the revised manuscript in lines 333 - 334, 431 - 432). We believe this part of the manuscript is of interest since it helps to consolidate a plausible mechanism of action that fits together with the transcriptional and chromatin data, and that could explain the observed defence phenotypes for *eda16* mutants. Finally, our ChIP data supports the notion of a direct involvement of EDA16 in regulating genes that could be linked with redox homeostasis.

- The observation that ETI is not affected by EDA16 is interesting and given its established role in MTI regulation, not surprising. In that context, I would encourage the authors to speculate as to why this could be. One hypothesis that could be put forward is that ETI often features (and requires?) drastic responses to pathogen ingress, eliminating the need for the dampening of responses. Alternatively, other modelling factors could be at play.

We thank the reviewer for the constructive comment. This is indeed a very interesting point that we are glad to discuss. We have only chosen not to delve on it since future research will need to help clarify how MTI and ETI may make the most of *EDA16* mechanism in particular and in general in recent years novel research is filling the gap to understand the interplay between MTI and ETI. We have now added to the Discussion (lines 405 – 408).
